# Association between homocysteine level and blood pressure traits among Tibetans: A cross-sectional study in China

**DOI:** 10.1097/MD.0000000000016085

**Published:** 2019-07-05

**Authors:** Pengfei Sun, Qianqian Wang, Yan Zhang, Yong Huo, Nima Nima, Jun Fan

**Affiliations:** a Department of Cardiology, Peking University First Hospital; b Department of Molecular Orthopaedics, Beijing Institute of Traumatology and Orthopaedics; c Department of Cardiology, Lhasa People's Hospital, Lhasa, Xizang; d Department of Cardiology, Jishuitan Hospital, Beijing, China.

**Keywords:** blood pressure, high altitude, homocysteine, hypertension, Tibetan

## Abstract

Studies on hypertension (HTN) in Tibetans who live in high altitude areas are less and whether total homocysteine level (tHcy) is associated with blood pressure (BP) levels or HTN status in Tibetans is unknown.

A total of 1486 Tibetans with complete information from a cross-sectional survey conducted in Lhasa Chengguan County of Tibet were included in this study. Demographic data, self-reported history of disease, and life styles were collected using a questionnaire. Blood tHcy, creatinine, fasting plasma-glucose, total cholesterol, triglycerides, and BP were measured with equipment.

The median tHcy level of the whole population was 14.60 (13.17–16.50) μmol/L, and the prevalence of HTN was 26.99%. Regression models, adjusted for possible covariates, showed that an average increase of 1 lnHcy (log transformation of tHcy level) was associated with an increase of 3.78 mmHg of systolic BP (SBP, *P* = .011) and 3.02 mmHg of diastolic BP (DBP, *P* = .003). The prevalence of HTN, levels of SBP and DBP in the third (OR for HTN: 1.60, *P* = .026; *β* for SBP: 3.41, *P* = .004; *β* for DBP: 2.57, *P* = .002) and fourth (OR for HTN: 2.19, *P* < .001; *β* for SBP: 5.08, *P* < .001; *β* for DBP: 3.09, *P* < .001) quartile of tHcy level were higher than those in the first quartile.

THcy is associated with BP levels and HTN status among Tibetans. Both HTN management and tHcy level should be paid more attention in Tibetans.

## 1 Introduction

Cardiovascular disease (CVD) represents the leading cause of mortality in developed and many developing countries. Hypertension (HTN) is one of the most important classical risk factors for CVD.^[1,2]^ On account of the high prevalence and big contribution to CVD, it's of great significance to identify new risk factors of HTN for the purpose of primary prevention.

Hyperhomocysteinemia (HHcy) has been found to be related to increased risk of CVD and senile dementia.^[3,4]^ Furthermore, synergistic effect of high total Hcy (tHcy) level (defined as ≥10 μmol/L) and HTN to stroke is much larger than HTN alone.^[5]^ However, the progressive contributions of tHcy on blood pressure (BP) levels/HTN are still under debate. On the one hand, some investigations, for example, Hordaland Homocysteine Study and a case–control research (n = 350) by Tyrrell et al showed positive relations between tHcy and HTN/BP levels.^[6,7]^ More recently, 3 investigations directed by Li et al, Yucel et al, and Lu et al found that tHcy had relations with HTN/BP levels.^[8–10]^ On the other hand, some studies, for example, a study on young adult African Americans and a big cohort study conducted in Framingham, showed that the relations between tHcy and HTN/BP levels were not statistically significant.^[11,12]^

An interesting thing lies in the fact that this divergence may be ethnic related to some extent since the conclusions of studies with participants in different ethnics can be adverse.^[8,13,14]^ So, the associations between tHcy and blood pressure traits in different ethnics are worth investigating. Tibetan is a special ethnic group who inhabit the highest plateau in the world which means they are exposed to low oxygen and temperature environment,^[15]^ besides, they are Chinese but used to eating meat and drinking milk,^[16]^ which makes them different from residents of both western and eastern countries. High altitude is also found to be associated with BP levels.^[17]^ As a special ethnic living in the highest plateau, the prevalence rate of HTN in Tibetans was pointed to be high^[16]^ which making it more meaningful to investigate the associations between BP levels and tHcy. As far as we know, this study is the first one to investigate the relationships between BP traits and tHcy level among Tibetans. We aimed to reveal the relations between prevalence of HTN/BP levels and tHcy in Tibetans and offer a foundation for future studies which analyze the association between HTN and tHcy in different ethnics as well as altitude.

## 2 Methods

### 2.1 Study sample

Residents of Lhasa Chengguan community were recruited to do physical exam by posters or by invited phone calls, those aged over 18 years old, did physical exam between June 14th and June 30th were included in this study. Cluster sampling was used by consecutively including participants who came onsite in the first 2 weeks of the examination to avoid selection bias. A total of 1524 residents volunteered to join in this study, subjects without complete information (n = 13) or those who were not Tibetans (n = 25) were excluded. At last, a total of 1486 individuals were included in our analysis. All participants gave informed consent and the study was approved by the Ethics Committee of Lhasa people's hospital. The methods were carried out in accordance with relevant guidelines and regulations.

### 2.2 Data collection

Sex, age, education, salt intake, current smoking, and history of disease were collected by trained investigators using a designed questionnaire for this study. Current smoking was defined as smoking >1 cigarette per day for at least half a year. Over salt diet was defined as self-reported salty taste based on the questionnaire.

Weight and height were measured by DHM-200. Participants were asked to wear light clothes and take shoes off. Weight was measured to the nearest 0.1 kg; height was measured to the nearest 0.5 cm. Body mass index (BMI) was calculated as weight in kilograms divided by the square of the height in meters.

Seated BP was measured 3 times on the right arm with 2-minute intervals after at least 10-minutes of rest using OMRON HEM-7130. Systolic BP (SBP) and diastolic BP (DBP) used in the analysis were the averages of 3 determinants. HTN was defined as SBP ≥140 mmHg or DBP ≥90 mmHg or self-reported HTN history

Blood samples were collected in the morning from a vein in forearm into tubes containing EDTA. Participants were asked not to eat or drink at least 12 hours before taking blood samples. Plasma samples were separated within 30 minutes of collection and were stored at –20 °C. Fasting plasma glucose (FPG), total cholesterol (TC), triglycerides (TG), and plasma creatinine (Scr) were measured using automatic biochemical analyzer DIRUI CS-1200 (Beijing Mairun, China). Plasma tHcy was measured using electrochemiluminescence method. HHcy was defined as tHcy ≥10 μmol/L. Diabetes mellitus was diagnosed according to the WHO criteria^[18]^: FPG ≥7 μmol/L or from self-reported history. Hyperlipidemia was defined as TC ≥5.2 mmol/L or TG ≥1.7 mmol/L.

### 2.3 Statistical analysis

Statistical analyses were performed using Empower (R) (www.empowerstats.com, X&Y solutions, Inc. Boston, MA) and R (http://www.R-project.org). A 2-tailed *P* < .05 indicated statistically significant differences. Relationships of tHcy level with prevalence of HTN/BP levels were our main interests. Descriptive statistics were performed and categorical variables were represented as numbers and percentages, while continuous variables were expressed as means ± SD or the medians (first-third quartile) by tHcy quartiles. Group differences were estimated using one way ANOVA or Kruskal-Wallis test depending on distributions for constant variables and Pearson chi-square for categorical variables.

Relationship between tHcy level and prevalence of HTN was estimated using multivariate logistic regression model. Relationships between tHcy and BP levels were estimated using multivariate linear regression models. Sex, age, education (middle school or below, senior high school or above), over salt diet, BMI, current smoking, diabetes, hyperlipidemia, and Scr were adjusted in the regression models. *P* for trend was estimated by recoding tHcy quartile as a continuous variable, which was then put into multivariate linear regression models adjusting for the above covariates. A generalized additive model by using a spline smoothing function was applied to examine the relationships of tHcy level with SBP and DBP levels with adjusting for potential confounders.

## 3 Results

### 3.1 Baseline characteristics

A total of 1486 Tibetans were included in the analysis and their characteristics stratified by tHcy quartiles were showed in Table 1. The average levels of SBP and DBP were (117.90 ± 19.80) mmHg and (79.11 ± 12.96) mmHg, respectively. The median plasma tHcy level was 14.60 (13.17–16.50) μmol/L. Subjects suffered with HTN, diabetes, and hyperlipidemia accounted for 26.99%, 3.30%, and 29.07%, respectively. Among them, only 30.42% (n = 122) of HTN patients reported a history of HTN. Age, sex, ratio of over salt diet, plasma Scr, prevalence of hypertension, and tHcy levels, but not current smoking status, hyperlipidemia, diabetes, education status, or BMI were significant different among participants in different tHcy quartiles (*P* < .05).

**Table 1:**
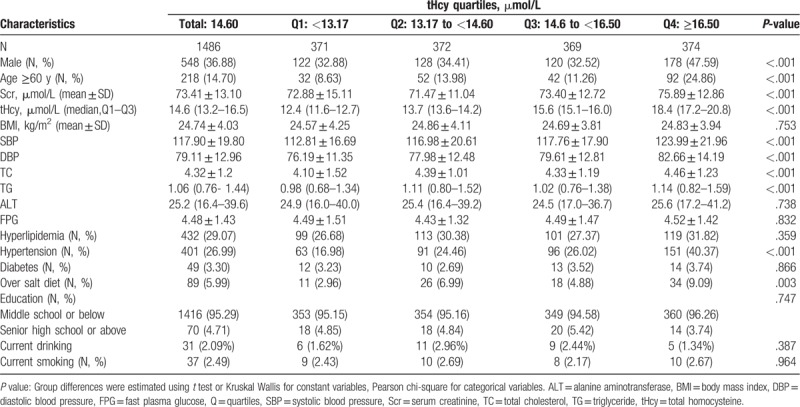
Baseline characteristics stratified by tHcy quartiles.

### 3.2 Associations between tHcy and levels of BP

**Figure 1:**
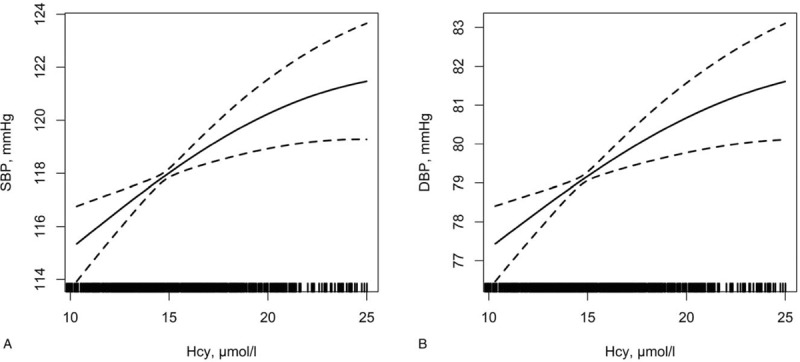
Association of SBP and DBP levels with tHcy. A: Association of SBP level with tHcy; B: Association of DBP level with tHcy. Adjusted for: age, BMI, sex, hyperlipidemia, over salt diet, Scr, education, smoking, and diabetes. BMI = body mass index, DBP = diastolic blood pressure, SBP = systolic blood pressure, tHcy = total homocysteine.

Table 2 shows the relationships between BP and tHcy levels. Figure 1 shows relationships between BP and tHcy levels after adjusting for possible covariates (sex, age, BMI, education, over salt intake, Scr, hyperlipidemia, current smoking, and diabetes). Logarithmic transformation value of tHcy was used in the analysis since tHcy was not consistent with normal distribution. An average increase of 1 lnHcy was associated with an increase of 8.31 mmHg of SBP (95% CI: 4.85–11.78, *P* < .001) and 5.56 mmHg of DBP (95% CI: 3.30–7.83, *P* < .001) in the crude model. These associations remained to be significant (for SBP: *β* = 3.78, 95% CI: 0.89–6.68, *P* = .011, for DBP: *β* = 3.02, 95% CI: 1.03–5.00, *P* = .003) even after adjusting for possible covariates. Furthermore, positive associations between SBP (*P* for trend <.001), DBP (*P* for trend <.001), and tHcy quartiles were observed in trend analysis. In the adjusted model, there was an increase of 3.41 mmHg (95% CI: 1.10–5.72, *P* = .004) of SBP in Q3 (the third tHcy quartile) and 5.08 mmHg (95% CI: 2.72–7.45, *P* < .001) in Q4 (the fourth tHcy quartile) compared with Q1 (the first tHcy quartile), respectively. Moreover, there was an increase of 2.57 mmHg (95% CI: 0.98–4.15, *P* = .002) of DBP in Q3 group and 3.09 mmHg (95% CI: 1.47–4.71, *P* < .001) in Q4 group compared with Q1 group. Subgroup analysis was also performed in participants who denied history of HTN to excluded influences of anti-hypertensive medications. Consistently, relations between tHcy quartiles and BP levels remained significant. There was an increase of 2.72 mmHg (95% CI: 0.47–4.97, *P* = .018) of SBP in Q3 group and 4.35 mmHg (95% CI: 2.05–6.65, *P* < .001) in Q4 group compared with Q1 group (*P* for trend <.001). There was an increase of 2.07 mmHg (95% CI: 0.50–3.63, *P* = .010) of DBP in Q3 group and 2.45 mmHg (95% CI: 0.85–4.05, *P* = .003) in Q4 group compared with Q1 group (*P* for trend <.001) after adjusting for the same covariates.

**Table 2:**
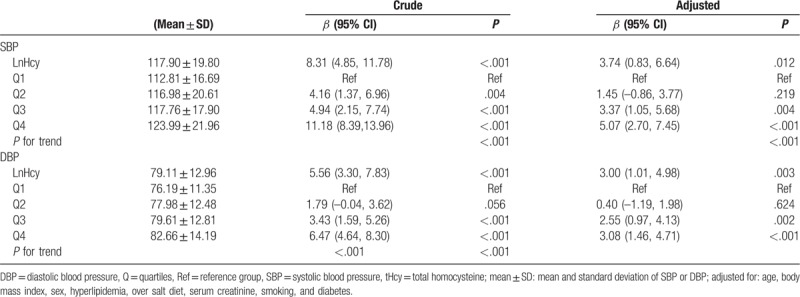
Association between tHcy and BP levels.

### 3.3 Association between tHcy levels and prevalence of HTN

Table 3 shows the relationships between tHcy levels and prevalence of HTN. The results showed that high tHcy levels were associated with increased HTN prevalence (*P* for trend <.001). Compared with Q1 group, prevalence of HTN was 1.60 (1.06, 2.42, *P* = .026) times higher in Q3 group and 2.19 (95% CI: 1.46–3.28, *P* < .001) times higher in Q4 group after adjusting for possible covariates.

**Table 3:**
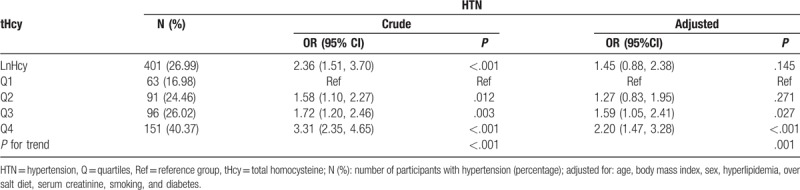
Association between tHcy and HTN status.

## 4 Discussion

Our study found positive relationships between BP traits and tHcy concentration in Tibetans. To our knowledge, this is the first demonstration of the association between tHcy level and BP traits in Tibetans.

Positive associations between BP traits and tHcy level were observed in our study. Several studies in Han populations also showed that tHcy level was associated with the prevalence of HTN or levels of BP. Momin demonstrated that lnHcy was associated with both central SBP (*β* = 2.17, *P* = .007) and brachial SBP (*β* = 2.42, *P* = .001) after adjusting for possible covariates in a community-based population in Beijing, China.^[19]^ A study conducted in Chinese hypertensive patients suggested that participants with higher baseline Hcy levels had persistently higher SBP level.^[20]^ Elsewhere, a cross-sectional study using data from the Third National Health and Nutrition Examination Survey (1998–1994) in Americans found that tHcy level was independently associated with BP levels after adjusting for cardiovascular risk factors. Every 1 standard deviation (similar to 5 μmol/L) increase in tHcy was associated with increases in SBP and DBP of 0.7 and 0.5 mmHg, respectively.^[21]^ Other cross-sectional studies including a study carried out in Tallinn consisting 511 men and 600 women with a mean age of 46 years^[22]^ and an investigation with 16,571 participants from western Norway also pointed positive associations between tHcy and BP traits.^[6]^ Moreover, similar results were obtained in case–control studies conducted in USA^[14]^ and Turkey.^[9,23]^ Folic acid supplementation resulted in a decrease of both plasma tHcy levels and BP levels can be another support for the relationship between tHcy level and HTN. In 2012, a cohort study including 4400 men and women aged 18 to 30 years found that higher folic acid intake in young adulthood was longitudinally associated with a lower incidence of HTN later in life, and this association was racial related.^[24]^

However, controversial results have been observed by several investigations. Framingham study with 2104 participants (mean age: 57 years; 58% of them were women) found that the relations of plasma tHcy with the incidence of HTN or levels of BP were statistically non-significant in age and sex adjusted models.^[12]^ Several cross-sectional studies also reported negative relationships between HTN/BP levels and tHcy level. Possible reasons for these discrepancies include small sample sizes, enrollment of participants from different ethnic origins, and adjusting for few covariates.^[11,25,26]^

There are several theories about the possible mechanism linking tHcy level and BP traits. THcy is believed to be associated with low bioavailability of nitric oxide, high oxidative stress, proliferation of vascular smooth muscle cells, and alteration of elastic properties of the vascular wall.^[27–29]^ Besides, endothelium damage due to HTN results in excessive release of Hcy particle to blood and then increases the level of this molecule in human blood.^[30]^

Tibetan is a Chinese minority population; data from 2010 China nationwide census showed that there was six million Tibetans in China and almost half of them lived in Tibet Plateau (the highest plateau in the world). Lhasa, with the altitude of 3650 m, the provincial capital of Tibet Autonomous Region, is an ideal place candidate for investigating the relationship between tHcy and BP levels among Tibetans. High prevalence of HTN and HHcy were observed in our study: the prevalence rate of HTN in our study was 26.99%; whereas that of HHcy in HTN participants was 98.00%. These findings were worthy for more attention based on the significant synergistic effect of HTN and HHcy on the new onset of stroke risk.^[5]^ Systematic management of HTN and HHcy in Tibetans is needed. Several unique factors such as attitude and dietary habit among Tibetans may affect BP and tHcy levels. High altitude has long been considered to be associated with BP levels.^[31]^ However, the association was discordant according to different populations. Aryal et al^[32]^ found that high altitude was associated with higher BP in participants of Tibetan origin but lower BP in participants of non-Tibetan origin. Calbet et al^[33]^ reported that short-term residence at high altitude increased arterial BP, reduced vasodilatory response to adenosine and ATP, which may be caused by chronic hypoxia. A study by Wu et al^[34]^ found that this short term elevation in BP returned to baseline levels within 3 months. High-sodium intake among Tibetans^[35,36]^ also influences their BP levels. Urinary sodium and sodium to potassium ratio excretion was higher in Tibetans than in Han subjects.^[36]^ Butter tea is a traditional and popular drink among Tibet Plateau inhabitants. It contains about 6 g/L salt^[37]^ and may be a possible reason for the high sodium intake. Besides, Tibetans tend to eat less vegetables and fruits which may result higher homocysteine level.^[24]^ Thus, the influence of altitude on the association between tHcy and BP level needs further research investigation.

This study demonstrated the association between tHcy level and BP traits in Tibetans firstly, since prevalence of HTN and HHcy are high in this survey, these findings were meaningful based on the significant synergistic effect of HTN and HHcy on new onset of stroke.^[5]^ More attention is needed for the management of HTN in Tibetans, given that almost one-third of participants in our study had HTN but only 30.42% of them reported a history of HTN. It has been shown that folic acid supplementation can reduce 21% risk of first stroke in addition of lowering BP among Chinese hypertensive adults.^[38,39]^ Our study highlights the feasibility of folic acid supplementation to achieve more benefits for stroke primary prevention in Tibetans in the future.

### 4.1 Limitations

There are several limitations in this study. First, this was a one-community based study, thus the results reported here may not be applicable to all Tibetans living in Lhasa and other different attitude levels. Nevertheless, our study is the first to demonstrate a positive association between tHcy and HTN in Lhasa Tibetans. Second, anti-hypertensive drugs information was not collected which may influence the association between tHcy and BP traits. The fact that the association remained statistically significant in participants without anti-hypertensive medications implies that these findings are robust. Third, the causal relationship between tHcy and BP traits remained unclear since our study is cross-sectional study.

### 4.2 Future directions

Cohort studies are needed, a Scheduled follow up in this population is on the way and will provide more evidences further. More investigations are needed for the influence of tHcy level as well as lowering tHcy level on incident hypertension.

## 5 Conclusions

Total Hcy level is associated with BP traits in our community-based Tibetan population. Due to high prevalence of combined HTN and HHcy among Tibetans, more investigations are needed for the influence of tHcy level as well as lowering tHcy level on incident hypertension.

## Acknowledgments

This study was supported by special funds for personnel resources development of Tibet Autonomous Region. They thank the support of Beijing Jishuitan hospital, all study team members of Lhasa people's hospital and Chengguan County.

## Author contributions

**Conceptualization:** Yong Huo, Nima Nima, Jun Fan.

**Data curation:** Pengfei Sun, Qianqian Wang.

**Formal analysis:** Pengfei Sun.

**Investigation:** Qianqian Wang, Yan Zhang, Yong Huo, Nima Nima, Jun Fan.

**Methodology:** Yan Zhang, Nima Nima, Jun Fan.

**Project administration:** Jun Fan.

**Resources:** Jun Fan.

**Writing – original draft:** Pengfei Sun.

**Writing – review & editing:** Qianqian Wang, Yan Zhang, Yong Huo, Nima Nima, Jun Fan.
